# The relationship between problematic smartphone use and dry eye disease symptom severity among Qatar University students: A cross-sectional study

**DOI:** 10.1016/j.pmedr.2026.103486

**Published:** 2026-05-02

**Authors:** Usra Elshaikh, Diyaa Rachdan, Khaloud Al-Marri, Maha Al-Qashoti, Hissa Al-Zoqari, Alya Naqadan, Raghad Saeed, Hanan F. Abdul Rahim, Mujahed Shraim

**Affiliations:** a Department of Public Health, College of Health Sciences, QU Health, Qatar University, Doha, Qatar; b College of Medicine, QU Health, Qatar University, Doha, Qatar; c Weill Cornell Medicine-Qatar, Cornell University, Doha, Qatar; d Ophthalmology Division, Department of Surgery, Sidra Medicine, Doha, Qatar

**Keywords:** Problematic smartphone use, Dry eye disease symptoms, University students, Cross-sectional study, Survey

## Abstract

**Objective:**

To investigate the association between problematic smartphone use (PSPU) and dry eye disease (DED) symptom severity among university students.

**Methods:**

A cross-sectional online survey was conducted in February – March 2021 among Qatar University students. The Smartphone Addiction Scale assessed PSPU, and the Ocular Surface Disease Index measured DED symptom severity. Bivariable and multivariable ordinal logistic regression models were constructed to examine the association.

**Results:**

A total of 157 students participated in the study (mean age: 22.7 years; 74.5% female). Nearly 74% of participants exhibited PSPU. Severe DED symptoms were more common in students with PSPU (27.6%) than in those without (12.2%). After adjusting for confounders, PSPU was associated with 2.29 times the odds of being in a higher DED symptom severity category (95% CI: 1.14, 4.58). For every one-point increase in the PSPU score, the odds of having a more severe category of DED symptoms increased by 4.00% (95% CI: 1.00%, 7.00%).

**Conclusions:**

Problematic smartphone use may be linked to greater DED symptom severity among university students. These findings suggest it may be valuable to consider smartphone use habits in clinical assessments and public health initiatives aimed at alleviating the burden of DED in this population.

## Highlights

• Problematic smartphone use is highly prevalent among university students.
• Problematic smartphone use is associated with dry eye disease symptoms severity.
• Integrating smartphone use behavior into clinical assessment of dry eye is important.
• Raising awareness of problematic smartphone use may help prevent dry eye symptoms.

## 1 Introduction

Dry eye disease (DED) is characterized by a multifactorial disorder of the ocular surface, resulting in symptoms of discomfort, visual disturbance, and tear film instability with potential damage to the ocular surface (Craig et al., 2017). Global prevalence estimates of DED vary widely, ranging from approximately 5% to 50%, depending on the population studied and the diagnostic criteria applied (Craig et al., 2017; Stapleton et al., 2017; Papas, 2021). Overall, the prevalence of DED increases with age and is higher among women and individuals of Asian ethnicity (Craig et al., 2017). DED represents a significant public health problem due to its adverse effects on vision, work productivity, quality of life, and the subsequent substantial economic burden (Chan et al., 2021; Boboridis et al., 2023; Binyousef et al., 2021). However, evidence on DED in younger populations remains limited, as few population-based studies have specifically evaluated its burden among adolescents and young adults (Stapleton et al., 2017).

The etiology of DED is complex and multifactorial, involving non-modifiable factors such as age, sex, race, and ocular or systemic diseases as well as modifiable factors including contact lens wear, hormone replacement therapy, systemic medications' side effects, environmental exposures such as pollution, low humidity, use of air conditioning, and the use of visual display terminal (e.g., computers, tablets and smartphones) (Stapleton et al., 2017). Considering the widespread and increasing reliance on smartphones globally, prolonged smartphone screen exposure has become a major concern, as it has been linked to reduced blink rate, incomplete blinking, and accelerated tear film evaporation, all of which contribute to tear film dysfunction (Fjærvoll et al., 2022; Ousler et al., 2014). Nevertheless, the literature examining the relationship between smartphone use and DED signs and symptoms remains limited. A recent systematic review identified only four studies, all conducted in South Korea, three of which reported a positive association between daily smartphone use and increased DED symptoms or clinical signs (Al-Marri et al., 2021). Within this context, problematic smartphone use (PSPU), characterized by excessive smartphone use that interferes with daily functioning (Kuss et al., 2018; Panova and Carbonell, 2018), has gained attention as a potentially significant and underrecognized contributor to DED. In the Middle East, the number of studies investigating this association remains minimal, and their findings are mixed. For example, a cross-sectional study among university students in Saudi Arabia found no statistically significant relationship between PSPU and DED (Baabdullah et al., 2019). In contrast, a cross-sectional study among Moroccan adolescents found a positive association between PSPU and DED symptom severity (Bouazza et al., 2025). Given the limited and mixed evidence currently available, further research is needed to clarify whether PSPU contributes to the development or worsening of DED. Establishing this relationship in settings characterized by high digital device dependence is particularly important as it has significant implications on raising public awareness and informing targeted interventions to prevent or mitigate the potential impact of PSPU on ocular surface health. Therefore, this study aims to investigate the relationship between PSPU and DED symptom severity among university students.

## 2 Methods

### 2.1 Study design and population

A cross-sectional online survey was conducted in February – March 2021 among all registered Qatar University (QU) students. Qatar University is the largest institution of higher education in Qatar, with a student body of approximately 25,000 (75.0% female and 68.0% Qatari nationals). The study was approved by QU Institutional Review Board (approval number QU-IRB 1307-EA/20). After obtaining ethical clearance, an electronic invitation was distributed via university email to all registered students, and the survey remained open for four weeks.

Eligibility criteria included being a QU student aged 18 years or older and able to read Arabic or English. Students with a history of ocular surgery, chronic ocular surface disease unrelated to DED, active use of contact lenses, systemic diseases affecting tear production, or current use of any eye drops were excluded.

### 2.2 Measures

Data were collected using a structured, self-administered online questionnaire. The questionnaire collected information on sociodemographic and behavioral characteristics, PSPU, and DED symptom severity. The sociodemographic and behavioral characteristics included age, gender (female, male), nationality (Qatari, non-Qatari), academic program (undergraduate, graduate), year of study (1st, 2nd, 3rd, 4th or higher), college, employment status (not employed, employed part-time, employed full-time), cigarette smoking (no, yes), frequency of performing at least 10 min of moderate or vigorous physical activity in the past week (none, 1–4 days, 5–7 days), history of physician-diagnosed chronic disease (no, yes), and the self-reported daily average duration (in hours) of smartphone and/or computer/tablet (including laptop) use and television viewing.

The primary exposure of interest was PSPU. PSPU is defined as excessive smartphone use that interferes with daily functioning (Kuss et al., 2018; Panova and Carbonell, 2018), which we operationalized using scores on the 10-item Smartphone Addiction Scale (SAS-SV). The SAS-SV, which has been validated in English and Arabic (Kwon et al., 2013; Sfendla et al., 2018), measures smartphone addiction-like symptoms across domains such as daily-life-disturbance, positive-anticipation, withdrawal, overuse, tolerance, and a cyberspace-oriented relationship (Kwon et al., 2013). Participants reported their agreement to statements on a 6-point Likert scale ranging from strongly disagree to strongly agree, yielding a total score between 10 and 60. Consistent with the scale's validation criteria, a participant was dichotomously categorized as having PSPU if their score was ≥33 for females and ≥ 31 for males (Kwon et al., 2013; Sfendla et al., 2018).

DED symptom severity was assessed using the Ocular Surface Disease Index (OSDI®), a widely used 12-item questionnaire that measures DED symptom severity, functional limitations, and environmental triggers over the previous week (Schiffman et al., 2000; Ozcura et al., 2007). Items were scored from 0 (“none of the time”) to 4 (“all of the time”). The OSDI total score was computed using the following standard formula:

$$\text{OSDI Total Score}=\frac{\left(\sum \text{of scores for}\;\mathrm{all}\;\text{questioned asnwerd}\right)\times 100}{\left(\text{Total number of questions answered}\right)\times 4}$$

All OSDI items were completed by every participant, with no missing responses. Scores were then classified into four severity categories: normal (0−12), mild (13−22), moderate (23−32), and severe (33−100) dry eye (Schiffman et al., 2000).

### 2.3 Statistical analysis

Data were analyzed using STATA 19 SE (StataCorp, 2025). Continuous variables were summarized using means and standard deviations (SD) or medians and interquartile ranges (IQR), while categorical variables were described using frequencies and percentages. Bivariable and multivariable ordinal logistic regression models were constructed to examine the relationships between PSPU, assessed both as a continuous variable and as a categorical variable (PSPU: no/yes), and OSDI severity categories. Variables demonstrating an association with DED symptom severity at a *p*-value of 0.20 or less in the bivariable analysis were included in the multivariable model to control for potential confounding (Heinze et al., 2018). The odds ratio (OR) and corresponding 95% confidence interval (CI) were used as measures of association. The proportional odds assumption of the ordinal logistic regression was evaluated using the Brant test (Brant, 1990). The proportional odds assumption was satisfied for all variables (Brant test, *p* > 0.05) except daily television watching duration (*p* < 0.01), and the global test indicated an overall violation of the assumption (*p* = 0.01). Therefore, a partial proportional odds model was fitted using the gologit2 command in Stata to relax the assumption for the violating variable (Williams, 2006).

## 3 Results

A total of 157 university students participated in the study (Table 1). Given that the total student population at Qatar University was approximately 21,000 in 2021, this corresponds to a response rate of approximately 0.8%, assuming all 21,000 met the eligible criteria, received, and read the invitations. The mean age of participants was 22.7 years (SD = 5.7). The majority were female (74.5%) and of non-Qatari nationality (70.7%). Most students were not employed (77.7%). Student representation varied by college, with the College of Arts and Sciences demonstrating the highest participation rate (24.2%), followed by the College of Engineering (18.5%) and the College of Medicine (15.3%). The majority were undergraduate students (82.8%), with a distribution across study years ranging from first year (36.3%) to fourth year or beyond (24.2%). (See Table 1.)

**Table 1:** Participant characteristics of students from Qatar University who participated in the survey, February – March 2021 (*n* = 157).

	Frequency (%)	Mean (SD); Median (IQR)
**Age**		22.7 (5.7); 21.0 (4.0)
**Gender**		
Female	117 (74.5)	
Male	40 (25.5)	
**Nationality**		
Qatari national	46 (29.3)	
Other nationalities	111 (70.7)	
**Employment status**		
Not employed	122 (77.7)	
Part-time	16 (10.2)	
Full-time	19 (12.1)	
**College**		
Arts and Science	38 (24.2)	
Business and Economics	7 (4.5)	
Education	12 (7.6)	
Engineering	29 (18.5)	
Law	6 (3.8)	
Health Sciences	21 (13.4)	
Medicine	24 (15.3)	
Pharmacy	11 (7.0)	
Sharia and Islamic Studies	9 (5.7)	
**Academic program**		
Undergraduate	130 (82.8)	
Graduate	27 (17.2)	
**Year of study in the program**		
1st	57 (36.3)	
2nd	30 (19.1)	
3rd	32 (20.4)	
4th or more	38 (24.2)	
**Smoking**		
No	147 (93.6)	
Yes	10 (6.4)	
**Physical exercise**		
None	73 (46.5)	
1–4 days	72 (45.9)	
5–7 days	12 (7.6)	
**Chronic disease status**		
No	142 (90.4)	
Yes	15 (9.6)	
**Daily smartphone use (hours)**		6.8 (3.2); 6.0 (4.0)
**Daily computer use (hours)**		5.1 (3.0); 5.0 (4.0)
**Daily TV viewing duration (hours)**		0.7 (1.2); 0.0 (1.0)
**Problematic smartphone use score**		38.9 (10.9); 41.0 (17.0)
**Problematic smartphone use status**		
No	41 (26.2)	
Yes	116 (73.9)	

SD, Standard Deviation; IQR, Interquartile range. Percentages may not total 100% due to rounding.

With respect to lifestyle characteristics, 93.6% of participants were non-smokers, and nearly half reported that they engaged in some level of physical exercise, with 45.9% reporting exercise frequency of 1–4 days per week and 7.6% reporting 5–7 days per week. Only 9.6% of participants reported having a chronic disease not related to tear dysfunction. The mean daily smartphone use was 6.8 h (SD = 3.2), while mean daily computer use was 5.1 h (SD =3.0), and television watching was relatively minimal with a mean of 0.7 h (SD = 1.2). The mean PSPU score was 38.9 (SD = 10.9), with nearly three-quarters (73.9%) classified as having PSPU. Overall, the median OSDI score was 18.8 (interquartile range (IQR) 8.3, 31.3). The median OSDI scores and IQRs for the DED symptom severity categories of Normal, Mild, Moderate, and Severe were 6.3 (2.1, 10.4), 18.8 (14.6, 20.8), 27.1 (25.0, 29.2), and 45.8 (39.6, 54.2), respectively.

### 3.1 The relationship between problematic smartphone use and dry eye symptom severity

Overall, participants with PSPU had a higher median OSDI score (20.8; IQR = 10.4, 35.4) than those without PSPU (12.5; IQR = 4.2, 22.9) (See Table 2). Among students with PSPU, 27.6% experienced severe dry eye symptoms, 15.5% had moderate symptoms, 25.9% had mild symptoms, and 31.0% had normal symptoms. In contrast, among students without PSPU, only 12.2% reported severe symptoms, 12.2% moderate symptoms, 22.0% mild symptoms, and 53.7% had normal symptoms.

**Table 2:** Crude and adjusted associations between problematic smartphone use and severity of dry eye symptoms among students from Qatar University, February – March 2021 (N = 157).

	Dry eye symptom severity				Crude association		Adjusted association *	
	Normal (*N* = 58)	Mild (*N* = 39)	Moderate (*N* = 23)	Severe (*N* = 37)	OR (95% CI)	SE	OR (95% CI)	SE
**Age (years), median (IQR)**	22.0 (6.0)	21.0 (4.0)	22.0 (3.0)	20.0 (3.0)	0.97 (0.92, 1.03)	0.03	–	–
**Gender, n (%)**								
Female	38 (32.5)	33 (28.2)	16 (13.7)	30 (25.6)	Ref		Ref	
Male	20 (50.0)	6 (15.0)	7 (17.5)	7 (17.5)	0.58 (0.30, 1.15)	0.20	0.63 (0.32, 1.26)	0.22
**Nationality, n (%)**								
Qatari national	11 (23.9)	15 (32.6)	5 (10.9)	15 (32.6)	Ref		Ref	
Other nationalities	47 (42.3)	24 (21.6)	18 (16.2)	22 (19.8)	0.54 (0.29, 1.00)	0.17	0.65 (0.32, 1.31)	0.23
**Employment status, n (%)**								
Not employed	44 (36.1)	32 (26.2)	16 (31.1)	30 (24.6)	Ref		Ref	
Part-time	4 (25.0)	4 (25.0)	3 (18.8)	5 (31.3)	1.57 (0.62, 3.98)	0.75	2.21 (0.85, 5.73)	1.07
Full-time	10 (52.6)	3 (15.8)	4 (21.1)	2 (10.5)	0.54 (0.22, 1.35)	0.25	0.76 (0.23, 2.48)	0.46
**College, n (%)**								
Arts and Science	14 (36.8)	10 (26.3)	7 (18.4)	7 (18.4)	Ref			
Business and Economics	3 (42.9)	1 (14.3)	0 (0.0)	3 (42.9)	1.36 (0.27, 6.77)	1.11	–	–
Education	5 (41.7)	6 (50.0)	0 (0.0)	1 (8.33)	0.59 (0.19, 1.83)	0.34	–	–
Engineering	10 (34.5)	8 (27.6)	2 (6.9)	9 (31.0)	1.25 (0.52, 3.01)	0.56	–	–
Law	1 (16.7)	3 (50.0)	0 (0.0)	2 (33.3)	1.66 (0.37, 7.51)	1.28	–	–
Health Sciences	6 (28.6)	4 (19.1)	5 (23.8)	6 (28.6)	1.64 (0.63, 4.24)	0.79	–	–
Medicine	13 (54.2)	4 (16.7)	2 (8.3)	5 (20.8)	0.61 (0.23, 1.61)	0.30	–	–
Pharmacy	3 (27.3)	2 (18.2)	3 (27.3)	3 (27.3)	1.68 (0.51, 5.53)	1.02	–	–
Sharia and Islamic Studies	3 (33.3)	1 (11.1)	4 (44.4)	1 (11.1)	1.24 (0.35, 4.36)	0.80	–	–
**Academic program, n (%)**								
Undergraduate	45 (34.6)	33 (25.4)	18 (13.9)	34 (26.2)	Ref		Ref	
Graduate	13 (48.2)	6 (22.2)	5 (18.5)	3 (11.1)	0.55 (0.26, 1.18)	0.21	0.68 (0.25, 1.82)	0.34
**Year of study in the program, n (%)**								
1st	24 (42.1)	9 (15.8)	9 (15.8)	15 (26.3)	Ref			
2nd	12 (40.0)	10 (33.3)	1 (3.3)	7 (23.3)	0.82 (0.36, 1.86)	0.34	–	–
3rd	11 (34.4)	8 (25.0)	5 (15.6)	8 (25.0)	1.13 (0.51, 2.49)	0.46	–	–
4th or more	11 (29.0)	12 (31.6)	8 (21.1)	7 (18.4)	1.13 (0.54, 2.36)	0.42	–	–
**Smoking, n (%)**								
No	55 (37.4)	37 (25.2)	22 (15.0)	33 (22.5)	Ref			
Yes	3 (30.0)	2 (20.0)	1 (10.0)	4 (40.0)	1.78 (0.53, 5.91)	1.09	–	–
**Exercise, n (%)**								
None	27 (37.0)	16 (21.9)	9 (12.3)	21 (28.8)	Ref			
1–4 days	27 (37.5)	21 (29.2)	13 (18.1)	11 (15.3)	0.76 (0.42, 1.37)	0.23	–	–
5–7 days	4 (33.3)	2 (16.7)	1 (8.3)	5 (41.7)	1.50 (0.47, 4.84)	0.90	–	–
**Chronic disease, n (%)**								
No	54 (38.0)	35 (24.7)	21 (14.8)	32 (22.5)	Ref			
Yes	4 (26.7)	4 (26.7)	2 (13.3)	5 (33.3)	1.62 (0.62, 4.24)	0.80	–	–
**Daily computer/tablet use (hours), median (IQR)**	4.8 (6.0)	6.0 (4.0)	5.0 (3.0)	4.0 (5.0)	1.00 (0.91, 1.10)	0.05	–	–
**Daily TV viewing duration (hours), median (IQR)**	0.0 (1.0)	0.5 (1.0)	0.0 (1.0)	1.0 (2.0)	1.33 (1.04, 1.72)	0.17	1.29 (0.98, 1.70)	0.18
**Problematic smartphone use status, n (%)**								
No	22 (53.7)	9 (22.0)	5 (12.2)	5 (12.2)	Ref		Ref	
Yes	36 (31.0)	30 (25.9)	18 (15.5)	32 (27.6)	2.53 (1.29, 4.97)	0.87	2.29 (1.14, 4.58)	0.81
**Problematic smartphone use score, median (IQR)**	35.5 (18.0)	44 (16.0)	41 (15.0)	45 (15.0)	1.04 (1.01, 1.07)	0.01	1.04 (1.01, 1.07)	0.01

OR, Odds ratio; CI, Confidence Interval; SE, Standard Error; Ref, Reference category; *DED symptom severity was adjusted for gender, nationality, employment status, academic program, and Daily TV viewing duration. An odds ratio > 1 presents higher odds of being in a more severe DED symptom severity category. Percentages are calculated based on the total for each row.

In the bi-variable analysis, students with PSPU had 2.53 times higher odds of greater DED symptom severity category compared with students without PSPU (95% CI 1.29, 4.97). After adjusting for gender, nationality, employment status, academic program, and daily television watching hours, the association between PSPU and DED symptom severity remained statistically significant (OR = 2.29, 95% CI 1.14, 4.58). After relaxing the proportional odds assumption for daily television watching duration using the partial proportional odds model, the association between PSPU and DED symptom severity remained essentially unchanged (OR = 2.29, 95% CI 1.14, 4.59). Similarly, when examined as a continuous variable, each one-point increase in PSPU score was associated with 4.00% higher odds of DED symptom severity category (adjusted OR = 1.04, 95% CI 1.01, 1.07) (Table 2).

## 4 Discussion

The present study examined the association between PSPU and DED symptom severity among university students. The findings showed that PSPU was associated with higher odds of DED symptom severity. These findings contribute to a growing body of evidence suggesting that PSPU represents an emerging behavioral predictor of DED symptoms, particularly among young populations. Our results are consistent with recent studies reporting statistically significant associations between PSPU and DED symptoms. In a cross-sectional study of Turkish university students, individuals classified as smartphone-addicted had significantly OSDI scores (median = 25.0, IQR = 12.0–45.8) compared with their non-addicted counterparts (median = 16.7, IQR = 6.2–33.3) (Ozalp, 2025). Similarly, a cross-sectional study among Moroccan high-school students reported that PSPU was positively associated with higher DED symptom scores, as measured using the Dry Eye Questionnaire-5 (*r* = 0.66) (Bouazza et al., 2025). Likewise, an earlier cross-sectional study among Korean university students reported a positive association between PSPU and OSDI scores (*r* = 0.33) (Paek, 2017).

In contrast, these findings differ from those of an earlier cross-sectional study among Saudi university students, which reported no statistically significant association between PSPU and DED symptoms measured using the OSDI scale (Baabdullah et al., 2019). This discrepancy may be partly attributable to differences in eligibility criteria, as the Saudi study included participants with established ocular and systemic risk factors for DED, whereas such individuals were excluded in the our study to minimize confounding. The inclusion of strong clinical risk factors may have attenuated the independent association between PSPU and DED symptoms, thereby contributing to the null findings reported in that study.

Several plausible mechanisms may explain the association between PSPU and increased DED symptoms. Smartphone use at a lower gaze angle, combined with sustained cognitive attention, has been associated with reduced blink rate, increased incomplete blinks, decreased tear volume, and shortened tear break-up time (Jaiswal et al., 2019). Additionally, smartphones are typically viewed at closer distances than other digital devices. Closer viewing however, has been associated with increased eyestrain and visual discomfort, suggesting higher accommodative and convergence demands during prolonged use (Long et al., 2017). The symptoms arising from excessive evening exposure to smartphone might become even worse due to the expected increase in symptoms of DED alone in the evening (Guillon and Shah, 2019). Another potential mechanism is that evening exposure to smartphone screen light may alter melatonin secretion and circadian rhythms while increasing stress hormone levels, thereby leading to poor sleep quality and disruption of tear secretion (Green et al., 2017; Galor et al., 2023). In addition, recent evidence suggests that sleep disturbance may partially mediate the relationship between PSPU and DED symptoms (Bouazza et al., 2025).

Our findings may have important public health and clinical implications. Early identification of individuals with PSPU could facilitate targeted screening for DED symptoms, particularly among populations and settings characterized by intensive smartphone use. Behavioral interventions, including scheduled screen breaks, blink reminders, and reduced nighttime screen exposure, may help mitigate DED symptoms (Sheppard and Wolffsohn, 2018). Furthermore, incorporating PSPU assessment into digital health promotion initiatives might be a strategy to reduce DED symptoms, especially among adolescents and young adults. In clinical practice, it may be worthwhile for ophthalmic clinicians to consider exploring smartphone use behaviors, alongside taking note of overall screen time, when evaluating patients presenting with unexplained dry eye complaints. In addition, public health initiatives that promote healthy digital habits could play a role in efforts to reduce the population-level burden of DED symptoms amid the growing prevalence of PSPU and DED.

A primary strength of our study is the examination of the association between PSPU and DED symptoms while accounting for important potential confounders, such as daily television viewing duration, and the use of validated instruments, including the SAS-SV and the OSDI, within a clearly defined population.

Several limitations should nevertheless be acknowledged. *First*, PSPU was self-reported and may be subject to recall or social-desirability bias. However, such bias is likely to underestimate rather than overestimate the relationship between PSPU and DED symptoms. *Second,* DED symptoms assessment was based on self-report and was not corroborated by objective clinical assessments, and symptom-based measures may not fully reflect clinical symptoms or severity. *Third,* several key potential confounding factors were not measured. Most notably, we did not collect data on total near work (activities involving focusing the eyes at a short distance), sleep quality, or mental health status, which are important factors that could have influenced the observed association. Additionally, environmental exposures such as humidity, air pollution, dust, and air conditioning, were not assessed. *Fourth*, this study was conducted while the world was recovering from the COVID-19 pandemic, which resulted in two important behavioral changes of relevance to the study. The first is the use of masks and hence the association with mask-associated dry eye and the second is the increased dependency on distance learning which by default increased the time spent using screens during the pandemic and thereafter (Cartes et al., 2022; Park et al., 2025). *Fifth,* due to the cross-sectional nature of the study, the possibility of reverse causation cannot be ruled out. It is plausible that individuals with pre-existing ocular discomfort might develop PSPU patterns as a form of digital distraction, avoidance coping, or for mood regulation. Longitudinal research is required to disentangle the directionality of this association. *Finally,* the relatively small sample of university students may limit generalizability to broader populations, including older adults or those with pre-existing eye disease. Therefore, these results should be interpreted with caution and require confirmation in larger cohort studies with diverse populations.

## 5 Conclusion

This cross-sectional study showed that problematic smartphone use is associated with greater dry eye disease symptom severity among university students. Although a causal relationship cannot be established, these findings suggest the potential value of considering smartphone use behaviors in clinical evaluations. Furthermore, this association may be a relevant factor for public health strategies aimed at understanding and addressing the burden of dry eye disease symptoms.

## CRediT authorship contribution statement

**Usra Elshaikh:** Writing – review & editing, Writing – original draft, Formal analysis, Data curation. **Diyaa Rachdan:** Writing – review & editing, Methodology, Conceptualization. **Khaloud Al-Marri:** Writing – review & editing, Data curation. **Maha Al-Qashoti:** Writing – review & editing, Data curation. **Hissa Al-Zoqari:** Writing – review & editing, Data curation. **Alya Naqadan:** Writing – review & editing, Data curation. **Raghad Saeed:** Writing – review & editing, Data curation. **Hanan F. Abdul Rahim:** Writing – review & editing, Supervision, Funding acquisition, Data curation, Conceptualization, Methodology. **Mujahed Shraim:** Writing – review & editing, Writing – original draft, Supervision, Project administration, Methodology, Investigation, Funding acquisition, Formal analysis, Data curation, Conceptualization.

## Funding

This research was funded by Qatar National Research Fund, Undergraduate Research Experience Program (UREP26-074-3-026). The funder had no role in study design; collection, analysis, and interpretation of data; writing the report; and the decision to submit the report for publication.

## Declaration of competing interest

The authors declare that they have no known competing financial interests or personal relationships that could have appeared to influence the work reported in this paper.

## Data Availability

Data will be made available on request.

## References

[bb0005] Al-Marri K, Al-Qashoti M, Al-Zoqari H, et al. The relationship between smartphone use and dry eye disease. Medicine (Baltimore) 2021/09/24/ 2021;100(38):e27311. doi:10.1097/MD.0000000000027311. PMC10545216 34559146

[bb0010] Baabdullah A.M., Abumohssin A.G., Alqahtani Y.A., Nemri I.A., Sabbahi D.A., Alhibshi N.M. (2019). The association between smartphone addiction and dry eye disease: a cross-sectional study. J. Nat. Sci. Med..

[bb0015] Binyousef F.H., Alruwaili S.A., Altammami A.F., Alharbi A.A., Alrakaf F.A., Almazrou A.A. (2021). Impact of dry eye disease on work productivity among Saudi Workers in Saudi Arabia. Clin. Ophthalmol..

[bb0020] Boboridis KG, Messmer EM, Benítez-Del-Castillo J, et al. Patient-reported burden and overall impact of dry eye disease across eight European countries: a cross-sectional web-based survey. BMJ Open 2023/03/17/ 2023;13(3):e067007. doi:10.1136/bmjopen-2022-067007. PMC10030789 36931668

[bb0025] Bouazza S, Abbouyi S, Zarrouq B. Moderated mediation model of the association between problematic smartphone use and dry eye disease within Moroccan adolescents. Sci. Rep. 2025/07/13/ 2025;15(1):25334. doi:10.1038/s41598-025-11060-4. PMC12256619 40653510

[bb0030] Brant R. (1990). Assessing proportionality in the proportional odds model for ordinal logistic regression. Biometrics.

[bb0035] Cartes C., Segovia C., Salinas-Toro D. (2022). Dry eye and visual display terminal-related symptoms among university students during the coronavirus disease pandemic. Ophthalmic Epidemiol..

[bb0040] Chan C, Ziai S, Myageri V, Burns JG, Prokopich CL. Economic burden and loss of quality of life from dry eye disease in Canada. BMJ Open Ophthalmol 2021 2021;6(1):e000709. doi:10.1136/bmjophth-2021-000709. PMC8444260 34604535

[bb0045] Craig JP, Nichols KK, Akpek EK, et al. TFOS DEWS II definition and classification report. Ocul. Surf. 2017/07// 2017;15(3):276–283. doi:10.1016/j.jtos.2017.05.008. 28736335

[bb0050] Fjærvoll K, Fjærvoll H, Magno M, et al. Review on the possible pathophysiological mechanisms underlying visual display terminal-associated dry eye disease. Acta Ophthalmol. 2022/12// 2022;100(8):861–877. doi:10.1111/aos.15150. PMC9790214 35441459

[bb0055] Galor A., Britten-Jones A.C., Feng Y. (2023). TFOS lifestyle: impact of lifestyle challenges on the ocular surface. Ocul. Surf..

[bb0060] Green A., Cohen-Zion M., Haim A., Dagan Y. (2017). Evening light exposure to computer screens disrupts human sleep, biological rhythms, and attention abilities. Chronobiol. Int..

[bb0065] Guillon M., Shah S. (2019). Rationale for 24-hour management of dry eye disease: a review. Cont. Lens Anterior Eye.

[bb0070] Heinze G, Wallisch C, Dunkler D. Variable selection – a review and recommendations for the practicing statistician. Biom. J. 2018 2018;60(3):431–449. doi:10.1002/bimj.201700067. PMC5969114 29292533

[bb0075] Jaiswal S., Asper L., Long J., Lee A., Harrison K., Golebiowski B. (2019). Ocular and visual discomfort associated with smartphones, tablets and computers: what we do and do not know. Clin. Exp. Optom..

[bb0080] Kuss D.J., Kanjo E., Crook-Rumsey M., Kibowski F., Wang G.Y., Sumich A. (2018). Problematic Mobile phone use and addiction across generations: the roles of psychopathological symptoms and smartphone use. J. Technol. Behav. Sci..

[bb0085] Kwon M, Kim D-J, Cho H, Yang S. The smartphone addiction scale: development and validation of a short version for adolescents. PloS One 2013/12/31/ 2013;8(12):e83558. doi:10.1371/journal.pone.0083558. PMC3877074 24391787

[bb0090] Long J., Cheung R., Duong S., Paynter R., Asper L. (2017). Viewing distance and eyestrain symptoms with prolonged viewing of smartphones. Clin. Exp. Optom..

[bb0095] Ousler GW, Abelson MB, Johnston PR, Rodriguez J, Lane K, Smith LM. Blink patterns and lid-contact times in dry-eye and normal subjects. Clin. Ophthalmol. 2014/05/05/ 2014;8:869–874. doi:10.2147/OPTH.S56783. PMC4015796 24833893

[bb0100] Ozalp M. Effect of mobile phone addiction on hand disorder, eye health, fatıgue and cognitive failures. BMC Public Health Jul 14 2025;25(1):2452. doi:10.1186/s12889-025-22154-z. PMC12257657 40660127

[bb0105] Ozcura F, Aydin S, Helvaci MR. Ocular surface disease index for the diagnosis of dry eye syndrome. Ocul. Immunol. Inflamm. 2007 2007;15(5):389–393. doi:10.1080/09273940701486803. 17972223

[bb0110] Paek K. A convergence study the association between addictive smart phone use, dry eye syndrome, upper extremity pain and depression among college students. J. Korea Converg. Soc. 01/28 2017;8:61–69. doi:10.15207/JKCS.2017.8.1.061.

[bb0115] Panova T, Carbonell X. Is smartphone addiction really an addiction? J. Behav. Addict. 2018/06/01/ 2018;7(2):252–259. doi:10.1556/2006.7.2018.49. PMC6174603 29895183

[bb0120] Papas EB. The global prevalence of dry eye disease: a Bayesian view. Ophthalmic Physiol. Opt. 2021/11// 2021;41(6):1254–1266. doi:10.1111/opo.12888. 34545606

[bb0125] Park K.S., Lee B.J., Ang M.J. (2025). Investigating mask-associated dry eye and contributing factors in healthcare providers. Clin. Ophthalmol..

[bb0130] Schiffman RM, Christianson MD, Jacobsen G, Hirsch JD, Reis BL. Reliability and validity of the ocular surface disease index. Arch. Ophthalmol. 2000/05// 2000;118(5):615–621. doi:10.1001/archopht.118.5.615. 10815152

[bb0135] Sfendla A, Laita M, Nejjar B, Souirti Z, Touhami AAO, Senhaji M. Reliability of the Arabic smartphone addiction scale and smartphone addiction scale-short version in two different Moroccan samples. Cyberpsychol. Behav. Soc. Netw. 2018/05// 2018;21(5):325–332. doi:10.1089/cyber.2017.0411. 29762065

[bb0140] Sheppard A.L., Wolffsohn J.S. (2018). Digital eye strain: prevalence, measurement and amelioration. BMJ Open Ophthalmol..

[bb0145] Stapleton F, Alves M, Bunya VY, et al. TFOS DEWS II epidemiology report. Ocul. Surf. 2017 2017;15(3):334–365. doi:10.1016/j.jtos.2017.05.003. 28736337

[bb0150] StataCorp. Stata Statistical Software: Release 19. 2025 2025.

[bb0155] Williams R. (2006). Generalized ordered logit/partial proportional odds models for ordinal dependent variables. Stata J..

